# Peracetic Acid: A Practical Alternative to Formalin for Disinfection of Extracted Human Teeth

**DOI:** 10.3390/bioengineering8120217

**Published:** 2021-12-15

**Authors:** Riaz Ali, Justin Bartholomew, Randolph Coffey, Caroline Carrico, Todd Kitten, Parthasarathy Madurantakam

**Affiliations:** 1 Department of General Practice, School of Dentistry, Virginia Commonwealth University, Richmond, VA 23098, USA; riazmali1@gmail.com (R.A.); bartholomewjd@mymail.vcu.edu (J.B.); rcoffey@vcu.edu (R.C.); 2 Department of Dental Public Health and Policy, School of Dentistry, Virginia Commonwealth University, Richmond, VA 23098, USA; ckcarrico@vcu.edu; 3 Philips Institute for Oral Health Research, Virginia Commonwealth University, Richmond, VA 23098, USA; tkitten@vcu.edu

**Keywords:** extracted teeth, disinfection, peracetic acid, formalin

## Abstract

Extracted human teeth provide the closest approximation to teeth in situ and play important roles in dental education and materials research. Since extracted teeth are potentially infectious, the Centers for Disease Control recommend their sterilization by autoclaving or disinfection by formalin immersion to ensure safe handling. However, autoclaving is not recommended for teeth with amalgam fillings and formalin is hazardous. The goal of the present study was to investigate the potential of peracetic acid (PA) as an alternative method to achieve reliable disinfection of freshly extracted teeth. A total of 80 extracted teeth were collected for this study. Whole teeth were incubated in one of four solutions for defined periods of time: sterile water (2 weeks), formalin (2 weeks), PA 1000 ppm (2 weeks), and PA 2000 ppm (1 week). After sectioning, the crown and root fragments were transferred into separate tubes containing brain–heart infusion broth and incubated at 37 °C under anaerobic conditions for 72 h. Absence of broth turbidity was used to assess effectiveness of disinfection. No turbidity was observed in any of the formalin-treated or peracetic acid-treated samples, signifying complete disinfection. Our results indicate that PA can effectively disinfect extracted human teeth, providing a reliable alternative to formalin and autoclaving.

## 1. Introduction

Extracted human teeth have been serving the profession of dentistry by providing a medium for training students in predoctoral programs, as well as acting as a test substrate on which new dental biomaterials are developed. An important component of predoctoral education in dental schools across the globe is the use of simulations. Students typically spend most of their preclinical years in simulation training to develop fine motor skills and apply learned didactic knowledge. Extracted human teeth have played an important role in these simulations because they possess natural tissue organization (enamel, dentin, pulp chamber and root canals) and physical attributes, including surface texture, cutting characteristic, hardness, color, radiopacity etc. [1] and retain chemical properties of etch and bond. Furthermore, extracted teeth are donated (by patients) and their use in training involves little additional expense. Despite these advantages, extracted teeth present a risk of cross-infection and their long-term storage requires careful adherence to established protocols. In addition, being sourced from different patients, inherent variations are unavoidable, and this makes standardization a challenge, particularly in the context of examination and licensing [2,3]. In order to address these limitations, synthetic alternatives are being explored. Most synthetic alternatives are resin-based, allowing for some control over the physical properties and geometry; however, these are often simplistic, prohibitively expensive for routine use, and lack the hardness and feel of natural teeth [4]. Until technology evolves to produce synthetic teeth that faithfully mimic the properties of enamel and dentin, their widespread adoption and mass production is unlikely, making the use of extracted human teeth inevitable. 

Over the past 3 decades, the concept of adhesive dentistry has revolutionized contemporary dental practice and has greatly widened the range of treatment options available. Adhesive dentistry refers to dental procedures and techniques that depend on true adhesion to tooth structure, rather than on traditional mechanical factors [5]. It is safe to say that none of these advances would have been possible were it not for laboratory studies using extracted human teeth. The in vitro assays evaluating bond strength, microleakage and marginal gap serve as fundamental screening tests that help predict clinical behavior of dental materials.

Since extracted teeth are designated by the Centers for Disease Control and Prevention (CDC) as potentially infectious [6] and most simulation exercises involving extracted teeth generate aerosols, reliable sterilization/disinfection techniques must be employed to ensure their safe handling. It has been clearly documented that extracted human teeth can support viable microorganisms and pose a risk of transmission through aerosols generated long after extraction [7]. In light of COVID-19 and heightened awareness of aerosol-transmissible diseases, it is imperative to minimize this risk.

The CDC recommends sterilization by autoclaving as the primary means to sterilize teeth without amalgam. For teeth with amalgam, immersion in 10% formalin solution for 2 weeks has been shown to disinfect the internal and external structures of the teeth [6]. Despite their effectiveness, both methods are associated with disadvantages. While autoclaving has been shown to adversely affect dentinal structure, including dentin permeability [8,9,10], formalin is a known cytotoxic and genotoxic agent [11]. Acute exposure to formalin can result in severe skin, eye, and respiratory tract irritation, while chronic exposure is associated with nasopharyngeal cancer and myeloid leukemia [12,13]. Being a respiratory irritant, formalin requires the use of fume hood, gloves, masks, and eye protection. Equally important are the environmental and legal regulations regarding its safe disposal. Given the drawbacks of these conventional methods, there has been a renewed interest in developing suitable alternatives to accomplish reliable disinfection of extracted human teeth for use in an educational or research setting [14]. 

Peracetic acid (PA) is a CDC-approved chemical sterilant that possesses excellent bactericidal, fungicidal, viricidal and sporicidal properties. PA, an equilibrium mixture of acetic acid and hydrogen peroxide, has been extensively used in the food industry because of its high potency and low residual toxicity. Furthermore, PA is effective at room temperatures, decomposes into nontoxic end products (water, oxygen, and carbon dioxide), and can be safely disposed of without affecting the environment [15]. These attributes make peracetic acid an interesting option to explore in the context of the disinfection of extracted teeth.

Our laboratory had previously demonstrated that PA treatment can be used to achieve sterilization of polymeric electrospun matrices [16]. Critical parameters were identified: diluent (20% ethanol), minimum concentration (1000 ppm), contact time (5 min), and temperature (20–25 °C). The present study was intended to evaluate if PA could be used to reliably disinfect fresh human teeth extracted due to chronic infection (gross decay or periodontitis).

## 2. Materials and Methods

All tooth extractions were carried out in the Department of Oral Surgery at Virginia Commonwealth University. Ten percent formalin (Sigma) was procured from the Department of Oral Pathology and used as one of the test solutions.

### 2.1. Extraction of Infected Teeth

All extracted teeth were collected in accordance with Virginia Commonwealth University, School of Dentistry guidelines and the research was carried out with the approval of the Virginia Commonwealth University Institutional Review Board (HM20022614). Since this study involved evaluation of disinfection, only teeth that were extracted due to pulpal or periapical infections were included in the study. Sound, non-carious teeth extracted for orthodontic reasons and impacted wisdom teeth were excluded. Twenty whole (non-sectioned) teeth were used for each of four disinfection treatments resulting in a total of 80 teeth. There was no exclusion based on upper or lower arch or location within the arch. 

### 2.2. Preparation of PA Solution

We prepared PA solutions as described previously [16]. Briefly, PA was purchased as a 39% solution (Sigma, St. Louis, MO, USA) in acetic acid and hydrogen peroxide. Different concentrations of PA (1000, 2000 ppm) were prepared by diluting the stock (390,000 ppm) in an appropriate volume of 20% ethanol. PA solutions were made fresh daily to ensure activity. 

### 2.3. Transfer of Extracted Teeth into Test and Control Solutions

Extracted teeth were transferred within 2 min directly into a disposable glass vial containing one of 4 solutions: water, formalin, 1000 ppm PA or 2000 ppm PA. The sterile water group served as a negative control for our experiments, while formalin immersion served as the positive control. The two concentrations of PA were the treatment groups of interest. The assignment of teeth to test solutions was randomized and the oral surgeon performing the extractions was blinded to the vial assignment. The immersion time of extracted teeth in test solutions were sterile water (2 weeks); formalin (2 weeks); PA 1000 ppm (2 weeks) and PA 2000 ppm (1 week). 

### 2.4. Sectioning of Teeth

All procedures described below were performed in a biosafety cabinet following strict sterile technique to prevent accidental contamination from the ambient environment. After the stipulated contact time for each of the four test solutions had elapsed, teeth were removed individually using a sterile forcep, rinsed thrice with autoclaved water and sectioned at the cemento–enamel junction using a sterile diamond bur to separate the crown and root fragments. 

### 2.5. Culture Conditions

Each crown and root section were then transferred into a separate tube containing 5 mL of sterile BHI (brain–heart infusion) broth (Sigma, St. Louis, MO, USA) and incubated with the lid loose in a jar at 37 °C under anaerobic conditions (80% N_2_, 9.9% CO_2_, 9.9% H_2_, and 0.2% O_2_, with the remaining O_2_ removed by a palladium catalyst) created with an Anoxomat Mark II system (Advanced Instruments, Norwood, MA, USA) for 72 h. 

### 2.6. Evaluation of Disinfection Effectiveness

**Figure 1:**
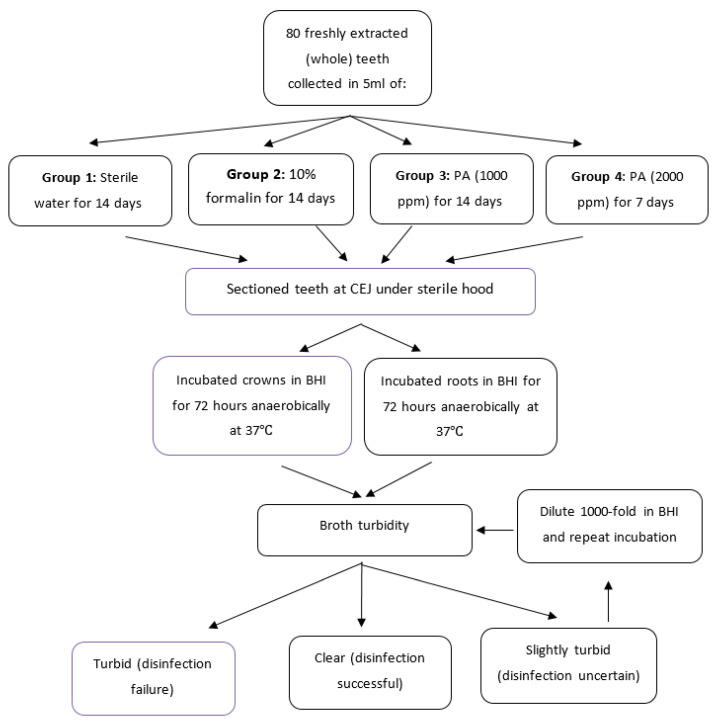
Research design.

After 72 h, the tubes were transferred into a sterile biosafety cabinet and the lids tightened. Each tube was then inverted several times and visually assessed for turbidity. Turbid broth would indicate bacterial growth and failed disinfection. If there was any ambiguity at this juncture (i.e., slight cloudiness/haze), the samples were diluted 1000-fold into fresh BHI and cultured for another 3 days to determine whether haziness was due to bacterial growth or was a result of suspended particles from the sectioning procedure. The overall scheme of the experimental design is presented in Figure 1. All tubes were evaluated by a single examiner after detailed calibration.

### 2.7. Sample Size Calculation 

When the sample size in each of the 4 groups is 20, a 0.05 level Chi-square test will have 86.5% power to detect an effect size of 0.16, which would reflect the scenario with 95% turbidity for sterile water group and 50% turbidity for each of the three experimental groups. 

### 2.8. Statistical Analysis 

Proportions of teeth that were disinfected (indicated by clear broth) were compared between sterile water and each sterilant group using Fisher’s exact test. A Bonferroni-adjusted significance level of 0.017 (0.05/3) was used for each Fisher’s exact test to account for multiple comparisons within crowns and roots. All analyses were performed using SAS EG v.8.2 (SAS Institute, Cary, NC, USA).

## 3. Results

The results of the experiments are presented in Table 1. All three treatment groups (formalin, PA 1000 ppm, and PA 2000 ppm) were significantly different from the sterile water group (*p* < 0.0001). There were no differences among the treatment groups. 

Sterile water was completely ineffective, with all samples (crown and root sections) producing turbidity in the BHI broth. In contrast, incubation of extracted teeth in formalin for 2 weeks achieved complete sterilization (0 of 40 tubes displaying turbidity). These results from positive and negative controls confirmed the validity of our procedures. 

A 2-week incubation in PA (1000 ppm) produced results comparable to formalin. Since the effectiveness of disinfection is a function of concentration of the disinfectant, as well as of contact time, we hypothesized increasing the concentration to 2000 ppm could be used to reduce the duration of disinfection. As shown in Table 1, PA at 2000 ppm achieved disinfection of all 40 samples in 1 week, potentially reducing the time to disinfect by as much as 1 week. 

## 4. Discussion

Extracted teeth have been indispensable in preclinical education in dental schools as well as in laboratory research on dental materials. Extracted human teeth are designated as potentially infectious by OSHA (Occupational Safety and Health Administration) and current CDC guidelines recommend autoclaving to sterilize teeth without amalgam and 2-week immersion in 10% formalin for teeth containing amalgam [6].

Sterilization using PA would avoid the disadvantages associated with autoclaving and formalin. While some studies show no effects after autoclaving, [17], others have shown significant reduction in dentin permeability (by 66%) due to collapse of collagen mesh [10] and changes in the chemical composition of dentin [18], thus limiting the applicability of results in experiments involving dentin. Moreover, the CDC recommends against sterilization of teeth containing amalgam fillings by autoclaving. 

There has been an ongoing search for an alternative to formalin to disinfect extracted teeth and a variety of methods have been described in the literature. Treatment with chemical disinfectants including quarternary ammonium chloride, [19] sodium hypochlorite, iodophors, glutaraldehyde and phenol failed to reliably disinfect the external tooth surface and the internal pulp tissue [20,21]. While ethylene oxide, [22] gamma irradiation, [23] and, more recently, vinegar [24] have been investigated for their ability to sterilize extracted teeth, most of these were associated with ineffective disinfection or adverse effects on enamel and dentin. [25,26,27] In this context, it is interesting to note that emerging studies have indicated that low concentrations of PA do not alter the radicular dentin [28] or negatively impact bond strength with self-adhesive cement, [29] or resin sealer [30].

There are many possible approaches to sterilant testing, including seeding of the test material with industry index microorganisms or spores [15]. Our previous published study explored sterilization of electrospun tissue engineering polymeric scaffolds using PA following intentional inoculation with *Bacillus* spores [16]. This was desirable because the engineered matrix was not contaminated after generation. In contrast, the intent of the present experiment was not to claim steril ization or high-level disinfection (as defined by the CDC). Instead, the goal was to develop a simple, reliable chairside method to disinfect naturally contaminated and infected teeth following their extraction. Hence, our choice of using pulpally/periodontally infected extracted teeth as our infection model instead of index microorganisms. We maintained the rigor of the microbial challenge by transferring teeth immediately into test solutions and avoiding the use of a storage medium. Furthermore, sectioning of the teeth after treatment allowed us to thoroughly investigate whether the internal, as well as external, surfaces of the teeth had been disinfected.

The limitations of the current study include using visual turbidity as an indicator of failed disinfection (as opposed to quantifying the turbidity using a spectrophotometer). We intentionally chose a strictly dichotomous variable as an outcome measure because disinfection is either successful or not. We considered any turbidity as failure of disinfection irrespective of the number of viable organisms. Another limitation is that our formulation presents unique challenges in dissecting the antimicrobial roles of individual components (peracetic acid, hydrogen peroxide and ethanol). Despite the above-mentioned issues, we believe that the study yielded clinically important information that can be routinely employed to disinfect teeth following extraction.

The ability of PA at 2000 ppm to achieve predictable sterilization in one week—half the time that we allotted to formalin and 1000 ppm PA—is an interesting finding. It is quite possible that PA achieves sterilization much earlier than 7 days; experiments are currently underway to investigate the kinetics of sterilization by including earlier timepoints. Future experiments will also investigate the effect of PA treatment on the enamel and dentin properties, especially in terms of bond strength and permeability.

## 5. Conclusions

Peracetic acid at concentrations of 1000 and 2000 ppm diluted in 20% ethanol presents a novel method of disinfecting human teeth extracted due to dental caries or periodontal infection. Peracetic acid can be used as an alternative to formalin to achieve reliable disinfection of extracted teeth in dental education settings.

## Figures and Tables

**Table 1:** Results of BHI cultivation of tooth fragments following the treatments indicated.

Treatment		Proportion of Tubes with Turbidity	
Sterilant	Time	Crown	Root
None (sterile H_2_O)	14 d	20/20	20/20
Formalin	14 d	0/20 *	0/20 *
PA 1000 ppm	14 d	0/20 *	0/20 *
PA 2000 ppm	7 d	0/20 *	0/20 *

* *p*-value < 0.0001 from Fisher’s exact test vs. sterile H_2_O.
